# Increased Expression of UMAMIT Amino Acid Transporters Results in Activation of Salicylic Acid Dependent Stress Response

**DOI:** 10.3389/fpls.2020.606386

**Published:** 2021-01-26

**Authors:** Julien Besnard, Unnati Sonawala, Bal Maharjan, Eva Collakova, Scott A. Finlayson, Guillaume Pilot, John McDowell, Sakiko Okumoto

**Affiliations:** ^1^Department of Plant Pathology, Physiology and Weed Science, Virginia Tech, Blacksburg, VA, United States; ^2^Department of Soil and Crop Sciences, Texas A&M AgriLife Research, Texas A&M University, College Station, TX, United States; ^3^School of Plant and Environmental Sciences, Virginia Tech, Blacksburg, VA, United States; ^4^Faculty of Molecular and Environmental Plant Sciences, Texas A&M University, College Station, TX, United States

**Keywords:** membrane transport, amino acid transport, amino acid metabolism, stress response, salicylic acid

## Abstract

In addition to their role in the biosynthesis of important molecules such as proteins and specialized metabolites, amino acids are known to function as signaling molecules through various pathways to report nitrogen status and trigger appropriate metabolic and cellular responses. Moreover, changes in amino acid levels through altered amino acid transporter activities trigger plant immune responses. Specifically, loss of function of major amino acid transporter, over-expression of cationic amino acid transporter, or over-expression of the positive regulators of membrane amino acid export all lead to dwarfed phenotypes and upregulated salicylic acid (SA)-induced stress marker genes. However, whether increasing amino acid exporter protein levels lead to similar stress phenotypes has not been investigated so far. Recently, a family of transporters, namely USUALLY MULTIPLE ACIDS MOVE IN AND OUT TRANSPORTERS (UMAMITs), were identified as amino acid exporters. The goal of this study was to investigate the effects of increased amino acid export on plant development, growth, and reproduction to further examine the link between amino acid transport and stress responses. The results presented here show strong evidence that an increased expression of UMAMIT transporters induces stress phenotypes and pathogen resistance, likely due to the establishment of a constitutive stress response via a SA-dependent pathway.

## Introduction

Plants absorb nitrogen (N) mostly in its two major inorganic forms, ammonium and nitrate ions. These ions are eventually assimilated into glutamine, then the N is transferred through transamination reactions to all other organic N molecules including other amino acids. In addition to the critical role as the central metabolites, amino acids serve as the N carrier between different organelles, tissues and organs. Distribution of assimilated N from the source (typically photosynthetic leaves) to sink tissues is mainly achieved through translocation of amino acids. To meet such needs, plant genomes encode numerous amino acid transporters (∼100 identified members of transporter families in Arabidopsis, Tegeder and Hammes, 2018), which function in transporting amino acids across the plasma membrane, as well as across organelle membranes.

Given the central role of amino acids in cellular metabolism and N balance between organs, it is not surprising that multiple mechanisms seem to exist to monitor the levels of amino acids. For example, TARGET OF RAPAMYCIN Complexes (TORCs) are cytosolic kinases that are activated by branched chain amino acids and are conserved among fungi, metazoans and plants (Nakajo et al., 2004; Fumarola et al., 2005; Kingsbury et al., 2015; Shimobayashi and Hall, 2016; Cao et al., 2019; Schaufelberger et al., 2019). Another cytosolic kinase, GENERAL CONTROL NON-REPRESSIBLE 2 (GCN2), is important for sensing N deficiency and is also conserved in fungi, metazoans, and plants. GCN2 does not sense amino acids *per se*, however it is activated when bound to de-acetylated tRNAs, which are more abundant when the cell is deprived of amino acids. Activated GCN2 represses protein synthesis by deactivating EUKARYOTIC ELONGATION INITIATION FACTOR 2α (eIF2α) through phosphorylation (Chantranupong et al., 2015). Therefore, TORC and GCN2 monitor the cytosolic amino acid sufficiency and deficiency, respectively. Additionally, GLUTAMATE RECEPTOR-LIKE (GLR) proteins, which structurally resemble the neuronal glutamate receptors from metazoans, seem to be involved in the perception of extracellular amino acids (Qi et al., 2006; Stephens et al., 2008; Vincill et al., 2012; Tapken et al., 2013; Kong et al., 2016; Goto et al., 2020). In addition to the proteogenic amino acids sensed by the above pathways, specialized non-proteogenic amino acids, such as β-aminobutyric acid (Luna et al., 2014) and pipecolic acid (PIP) and its derivative N-hydroxypipecolic acid (NHP) (Huang et al., 2020), function as defense signaling molecules, both of which activate the SA-dependent defense pathway.

The existence of amino acid sensing mechanisms in plants suggests that alteration of amino acid levels via altered amino acid metabolism or transport might trigger pleiotropic responses. Indeed, several independent studies in which the transport of amino acids has been enhanced or altered seem to confirm this possibility. A study using knockout lines for the amino acid importer LYSINE HISTIDINE TRANSPORTER 1 (LHT1) revealed that amino acid contents within the tissue and the extracellular fluid were both altered, and these mutants showed a constitutive stress response mediated by salicylic acid (SA) (Hirner et al., 2006; Liu et al., 2010). Similarly, overexpression of CATIONIC AMINO ACID TRANSPORTER 1 (CAT1) caused a stunted phenotype, associated with increased SA levels, resistance against *Pseudomonas synringae*, and the upregulation of genes associated with the development of systemic acquired resistance (pathogenesis- related genes or *PR*) (Yang et al., 2014). Similar observations were reported upon over-expression of GLUTAMINE DUMPER 1 (GDU1), a single-transmembrane protein which promotes amino acid export through an unknown mechanism (Pilot et al., 2004; Pratelli et al., 2010). Similar to *lht1* knockouts and CAT1 overexpressor mutants, SA levels in GDU1 overexpressing plants were elevated (Liu et al., 2010).

Recent discovery of UMAMITs which are bidirectional facilitators of amino acid transport, offer the possibility to interrogate the response of plants to increased amino acid export directly. The goal of this study was to investigate the effects of overexpressing UMAMIT genes that were shown to promote amino acid transport in plants (UMAMIT14, 18, 24, and 25, Ladwig et al., 2012; Müller et al., 2015; Besnard et al., 2016, 2018) on plant development, growth, and reproduction to further examine the link between amino acid transport and stress responses. The results presented here show strong evidence that amino acid export activity positively correlates with stress phenotypes and pathogen resistance, most likely due to the establishment of a constitutive SA-mediated stress response.

## Materials and Methods

### Plant Culture

Arabidopsis plants (Col-0) for observing phenotypes, mRNA and protein levels were grown in long days (16 h light at 50 μmol m^–2^ s^–1^ at the soil surface, 50% humidity, 22°C) in soil composed of 2:1 Sunshine Mix^TM^: vermiculite. Plants were watered with 0.15 g/L MiracleGro^TM^ fertilizer (24/8/16, N:P:K) three times a week. For *Hyaloperonospora arabidopsidis* infection, plants were grown under 8 h of light at 22°C and 16 h of dark at 20°C For kanamycin selection, seeds were sown on half-strength MS medium (1/2 Murashige and Skoog salt supplemented with 30 mM sucrose and 0.8% agarose with pH adjusted to 5.8 with KOH) containing 50 μg/ml kanamycin. Kanamycin-resistant plants were transferred to the long day conditions described above after 1 week of selection. Wild type Arabidopsis plants were transformed using the floral dip method using *Agrobacterium tumefaciens* strain GV3101 (pMP90) (Clough and Bent, 1998) to generate the overexpressor lines for UMAMIT14, UMAMIT18, UMAMIT24 and UMAMIT25.

### DNA Constructs

*UMAMIT14* genomic sequence (from ATG to the amino acid before the stop codon) was PCR-amplified from Col-0 genomic DNA with primers carrying *attb1* and *attb2* sequences flanking the genomic region. The PCR fragments were cloned into pDONRZeo vector using BP clonase II (Life Technologies, United States), and all entry clones were sequenced prior to use. *UMAMIT14* genomic sequence was transferred to the destination vector pPWYTkan (Besnard et al., 2016) using LR clonase II (Life Technologies, United States) to generate the 35S:UMAMIT14 lines. The same cloning steps were used for the creation of the lines over-expressing the genomic sequences of UMAMIT18, UMAMIT24, and UMAMIT25. A list of primers used for cloning is available in Supplementary Table 1.

### Protein Electrophoresis and Western Blotting

Leaf protein was extracted from rosette leaves of plants about 3-weeks old. The leaves were ground in liquid nitrogen, then vortexed for 5 min in the extraction buffer [250 mM Tris-HCl, pH. 8.5, 25 mM EGTA, 0.88 M sucrose, 5 mM DTT, Complete Proteinase Inhibitor (Roche, United States) at 1 tablet/50 mL volume], added at 3:1 v/w to the ground leaves. The soluble protein fraction was isolated from debris by centrifuging at 14,000 rpm for 10 min twice. Protein concentrations were determined using the Bradford assay (Coomassie Protein Assay Kit, Pierce, United States), following the procedure recommended by the manufacturer. Ten μg of extracted leaf proteins were denatured at 50°C for 15 min in an equivalent volume of loading buffer (62.5 mM Tris-HCl pH 6.8 adjusted with KOH, 2.7 M glycerol, 150 μM bromophenol blue and 70 mM SDS). Proteins were separated by SDS-PAGE and transferred to a Hybond^TM^ ECL (GE Healthcare, United Kingdom) nitrocellulose membrane following the manufacturer’s recommendations. UMAMIT14-c-myc was detected using anti-cmyc rabbit polyclonal IgG (Santa Cruz Biotechnology, clone sc-789; 1/4,000) and anti-rabbit IgG conjugated to horseradish peroxidase (1/10,000). Antibodies were detected by reaction with the ECL^TM^ Prime Kit (GE Healthcare, United Kingdom) using the manufacturer’s recommendations.

### RNA Extraction and qRT-PCR

RNA was extracted using the RNAeasy plant kit (Qiagen, United States) according to the manufacturer’s recommendation. Two μg of total RNA was used for cDNA synthesis with random primers using the iScript advanced cDNA synthesis kit (Bio-rad, United States). The list of primers used for qRT-PCR are described in Supplementary Table 1. QRT-PCR was performed using iTAQ Universal SYBR Green Supermix (Bio-rad), according to the manufacturer’s recommendation. Fold change relative to WT was calculated using the pcr package in R, using standard curves obtained with serial dilution of cDNA samples (Ahmed and Kim, 2018).

### Sporangiophore Assay and Trypan Blue Staining for *Hyaloperonospora arabidopsidis* Infection

The sporangiophore assay was performed using *Hyaloperonospora arabidopsidis* isolate Noco2 on 12-day-old seedlings. Trypan staining was performed to visualize hyphal growth and cell death on samples collected at 7 dpi (days post-inoculation). Both the sporangiophore assay and trypan staining were performed as described in Mcdowell et al. (2011).

### Analysis of Amino Acid Levels

Amino acid extractions and analyses in wild type and *35S:UMAMIT14-4* leaves were performed in wild type and *35S:UMAMIT14-4* leaves using Waters ultra-performance liquid chromatography coupled to fluorescent detection (UPLC-FLD) as described (Besnard et al., 2016; Schneider et al., 2016). Briefly, leaves of 5-week-old Arabidopsis plants were extracted after lyophilization and pulverization using biphasic extractions. Norvaline (0.5 μM final concentration) was used as an internal standard. Aqueous phase was derivatized using Waters AccQ∙Tag ^TM^ Ultra Amino Acid kit and injected on a Waters H-class Acquity UPLC-FLD equipped with a 10 cm Waters AccQ∙Tag^TM^ Ultra C18 column (1.7 μm, 2.1 mm × 100 mm; Waters, Milford, MA). Waters 10.2 min free amino cell culture chromatographic method was used to separate different amino acids (Waters). Data analysis was performed as described (Collakova et al., 2013).

### Quantification of Plant Hormones

SA, indole-3-acetic acid (IAA) and abscisic acid (ABA) were extracted and quantified using LC-MS/MS-based multiple reaction monitoring and isotope dilution based on the method of Liu and Finlayson, 2019). SA was quantified in negative ion mode monitoring the transitions 137.0–93.10 (endogenous) and 141.0–97.10 (labeled standard) with a cone voltage of 29 V and a collision energy of 16 eV.

### Statistical Analyses

One-way ANOVA followed by Tukey’s test, or two- tailed *t*-tests were used to determine significant differences (*p* < 0.05) between samples in Prism (Graphpad, United States).

## Results

### Expression of UMAMIT Amino Acid Exporters Induces a Stunted Phenotype in Arabidopsis

Previous studies have shown that *gdu1-1D* plants, in which amino acid export activity is enhanced, show pleiotropic stress phenotypes including smaller plant sizes and increased stress marker expression (Pilot et al., 2004; Liu et al., 2010; Pratelli et al., 2012; Besnard et al., 2016). In order to investigate a direct relationship between amino acid export and a stress phenotype, we sought to over-express an amino acid exporter. UMAMIT14, which functions as an exporter for charged (His, Glu, Asp), polar (Gln, Asn, Ser, Thr) and non-polar (Ala, Val, Ile, Leu, Phe) amino acids when expressed in yeasts, was chosen for the study (Müller et al., 2006; Besnard et al., 2016). Transgenic Arabidopsis lines that over-express *UMAMIT14-cmyc* under the control of the 35S promoter were constructed.

**FIGURE 1:**
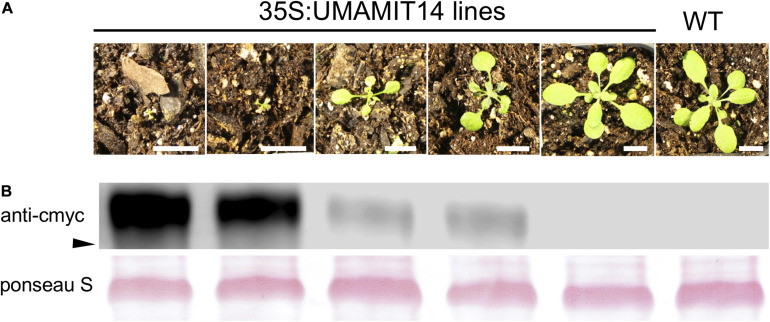
Constitutive expression of UMAMIT14 leads to a stunted phenotype. **(A)** Macroscopic phenotypes of 3-week-old Arabidopsis plants from T2 generation, expressing UMAMIT1-cMyc fusion under 35S promoter, compared to the wild type plant (right). Scale bar: 1 cm. **(B)** Top: Western blot targeting UMAMIT14-c-myc from corresponding lines shown in **(A)**. Each lane contained 10 μg of proteins extracted from 4-week-old wild type and 35S:UMAMIT14 leaves. Arrowhead indicates 40 kDa. Bottom: RubisCO protein visualized with Ponseau S staining of the membrane used for the Western blot shown in the top panel.

The observed phenotype was indeed reminiscent of *gdu1-1D*, showing stunted growth after 3 weeks (Figure 1A). The degree of dwarfism varied widely among the T1 and T2 plants, and for the individuals with most severe phenotypes we were unable to establish a homozygous line. Quantification of UMAMIT14-cMyc protein revealed that the phenotypic severity in T2 generation plants was positively correlated with the level of c-Myc-tagged UMAMIT14 protein expression (Figures 1A,B), similar to GDU1 over-expressors (Pilot et al., 2004). To further confirm that the phenotype is caused by amino acid export, three additional UMAMIT proteins, UMAMIT18, 24, and 25 (Ladwig et al., 2012; Besnard et al., 2016, 2018) were expressed under the control of the 35S promoter. UMAMIT14, 18, 24, and 25 share multiple substartes (Gln, Ala, Thr, Val, Ile), although the substrate specificity for other amino acids differ slightly; for example, Asp and GABA secretion is not increased in yeasts by UMAMIT14 expression, but is increased by UMAMIT24 and 25 (Besnard et al., 2016, 2018). Similar stunted phenotypes were observed for plants overexpressing UMAMIT 18, 24, and 25 (Supplementary Figure 1). Taken together, the results indicate that over-expression of UMAMIT transporters induces a pleiotropic phenotype including growth retardation.

**FIGURE 2:**
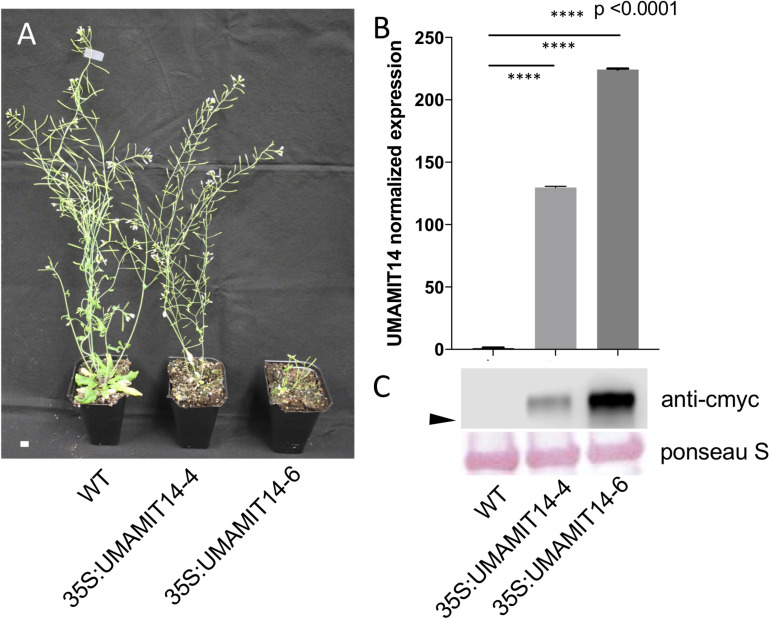
Phenotypes of 35S:UMAMIT14-4 and 14–6 lines. **(A)** Appearance of WT, 35S:UMAMIT14-4 and 35S:UMAMIT14-6 plants at maturity (12-weeks-old). Scale bar: 1 cm. **(B)** Relative *UMAMIT14* expression levels in 2-week-old seedlings of 35S:UMAMIT14-4 and 35S:UMAMIT14-6 lines compared to the wild type. Statistical significance at *p* < 0.05 is shown (asterisk) using one-way ANOVA and Tukey’s *post hoc* tests. Error bars: standard deviation, *n* = 4.

From the UMAMIT14 overexpressor lines, two representative lines, *35S:UMAMIT14-4 and 35S:UMAMIT14-6*, which show moderate and strong growth retardation phenotypes, respectively, were selected for further analysis (Figure 2A). *35S:UMAMIT14-4* and *35S:UMAMIT14-6* lines accumulated about 130- and 220-fold more *UMAMIT14* mRNA in leaves compared to the wild type, respectively, reflecting the severity of the phenotypes (Figure 2B). Western blot analysis showed that the difference in the mRNA amount between the lines is reflected in the amount of UMAMIT14 protein expressed (Figure 2C).

In addition to the stunted phenotype, *35S:UMAMIT14-4* and *35S:UMAMIT14-6* lines showed pleiotropic changes in various growth parameters. Under long day conditions, *35S:UMAMIT14-4* and *35S:UMAMIT14-6* displayed reduced plant biomass, silique length, and seeds per silique compared to the wild type (Table 1). In addition, plant height and seed weight were decreased in *35S:UMAMIT14-6* compared to the wild type.

**TABLE 1:** Characteristics of 9-week-old Arabidopsis plants grown in soil under long day conditions.

	Plant height (cm)	Biomass^∗^ (g)	Seed weight (mg)	Silique length (mm)	Seeds per silique
WT	34.6 ± 2.9 (a)	0.490 ± 0.021 (a)	94.32 ± 25.61 (a)	17.5 ± 1.0 (a)	58.7 ± 8.1 (a)
35S:UMAMIT14-1	28.7 ± 2.8 (a)	0.273 ± 0.116 (b)	104.84 ± 37.80 (a)	8.7 ± 1.5 (b)	16.7 ± 4.7 (b)
35S:UMAMIT14-4	3.6 ± 2.2 (b)	0.031 ± 0.007 (c)	16.28 ± 5.59 (b)	9.5 ± 1.7 (b)	25.6 ± 6.2 (c)

**Biomass represents all plant tissue collected from the aerial parts, minus the seeds. Significant differences (p < 0.05) are indicated by different letters according to one-way ANOVA followed by Tukey’s test (n = 4 biological replicates).**

### SA-Mediated Stress Responses Are Upregulated in *35S:UMAMIT14* Lines

**FIGURE 3:**
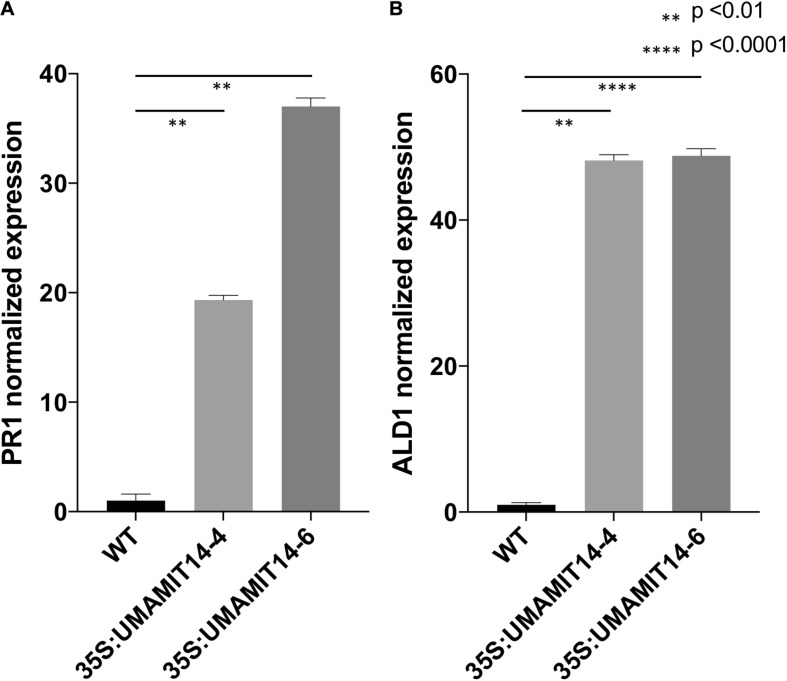
Relative expression levels of PR1 **(A)** and ALD1 **(B)** in 35S:UMAMIT14 lines. The expression levels of PR1 and ALD1 genes in 2-week-old 35S:UMAMIT14 plants were normalized to the expression levels in the wild type. Error bars: standard deviation, *n* = 4. Significant difference compared to the wild type are indicated by stars according to one-way ANOVA and Tukey’s *post hoc* tests.

**FIGURE 4:**
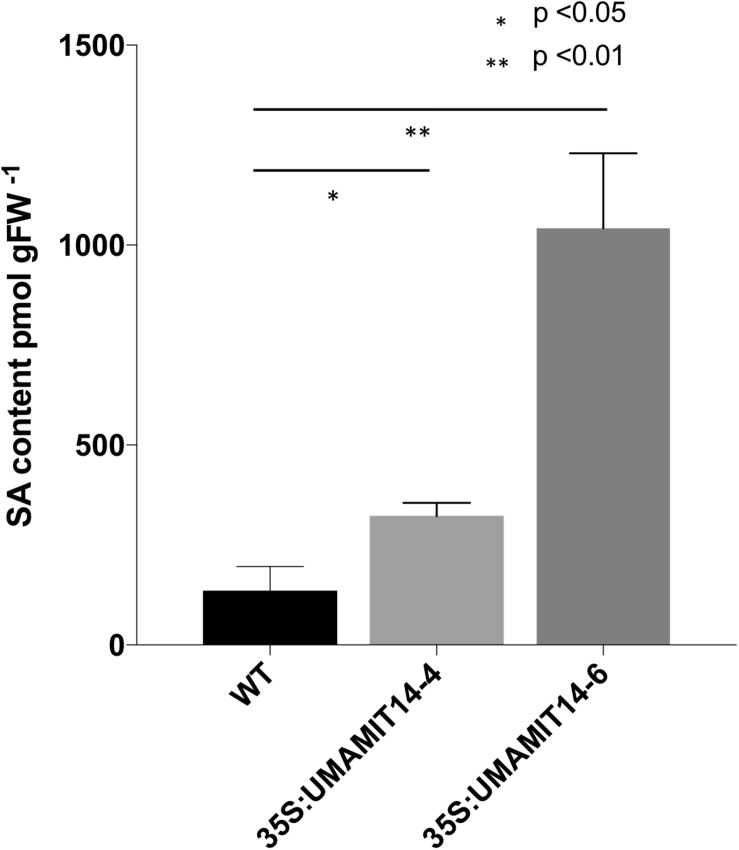
SA content in 35S:UMAMIT14-4 and 35S:UMAMIT14-6 plants. Total SA was extracted from the leaves of 3-week-old 35S:UMAMIT14-4 or 35S:UMAMIT14-6 plants. Student’s *t*-test was conducted to evaluate the statistical difference (indicated by an asterisk). Error bars: standard deviation, *n* ≥ 4.

Previous studies have shown that alteration of membrane amino acid transport induces SA-mediated stress responses (Liu et al., 2010; Yang et al., 2014). To evaluate whether similar responses are induced in overexpression lines, we quantified the expression of *PR1*, a marker gene for the SA pathway (Van Loon et al., 2006). *PR1* mRNA content was greatly increased in the leaves of both *35S:UMAMIT14-4* and *35S:UMAMIT14-6* plants (Figure 3A). In addition, the expression of *AGD2-LIKE DEFENSE RESPONSE PROTEIN 1 (ALD1)*, which encodes the enzyme catalyzing the first committed step of pipecolic acid (PIP) and NHP biosynthesis, was also increased in *35S:UMAMIT14-4* and *35S:UMAMIT14-6* plants (Figure 3B). The SA content in leaves of *35S:UMAMIT14-4* and *35S:UMAMIT14-6* plants was also significantly higher compared to the WT (Figure 4). No consistent trends for other plant hormones tested (IAA,ABA and JA) were found between *35S:UMAMIT14-4* and *35S:UMAMIT14-6* plants (Supplementary Figure 2).

### Transgenic Lines Overexpressing UMAMIT14 Gene Displayed Enhanced Disease Resistance

**FIGURE 5:**
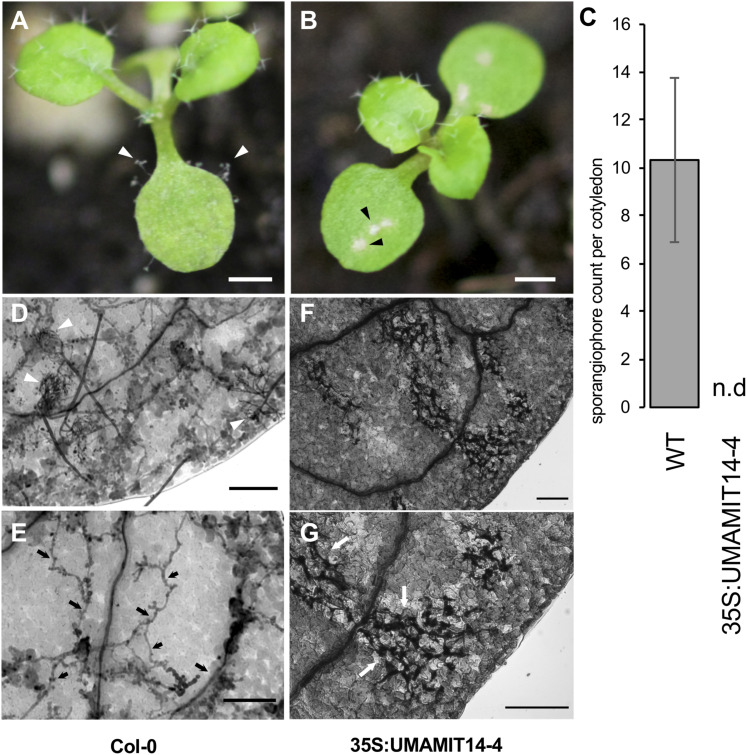
Responses of WT and 35S:UMAMIT14-4 to *Hyaloperonospora arabidopsidis* infection. **(A)** Col-0 (left) and **(B)** 35S:UMAMIT14 (right) inoculated with *Hyaloperonospora arabidopsidis* (Noco2), 7 days post-inoculation. Col-0 cotyledons display asexual reproductive structures (sporangiophores) whereas 35S:UMAMIT14 cotyledons display macroscopic lesions indicating cell death/necrosis. Scale bar: 1 mm. **(C)** Sporangiophore counts on 11-day-old cotyledons. The cotyledons were inoculated with 5 × 10^4^ spores per ml and counted after 7 day post-inoculation. N.d. no sporangiophores were detected on cotyledons. **(D–G)** Trypan blue-stained cotyledons of Col-0 **(D,E)** and 35S:UMAMIT14 **(F,G)** inoculated with *Hyaloperonospora arabidopsidis* (Noco2), 7 days post-inoculation. **(D)** Multiple sporangiophores (white arrows) are visible on the surface. **(E)** Hyphal growth (black arrows) are visible within the leaf tissue. **(F)** No sporangiophore is visible on the leaf surface. **(G)** Areas of cell death are visible by trypan blue staining (white arrows). Note the absence of hyphae within the leaf tissue. Scale bars: 100 μm.

We reasoned that if the SA-dependent defense pathway is activated in *35S:UMAMIT14* plants, their resistance to the pathogens known to activate the SA pathway would be bolstered (Mcdowell et al., 2011; Wei et al., 2015). Indeed, *35S:UMAMIT14-4* plants were found to be resistant to the biotrophic pathogen *Hyaloperonospora arabidopsidis* (*Hpa*), while the wild type remained sensitive. At 7 days post-inoculation, cotyledons of wild type plants developed sporangiophores whereas no sporangiophores were detected on *35S:UMAMIT14-4* plants (Figures 5A–D). The cotyledons of *35S:UMAMIT14-4* plants also showed macroscopic lesions caused by cell death, similar to those that have been observed in *gdu1-D* plants (Figure 5B; Liu et al., 2010). Hyphal growth within the leaf tissue is inhibited, concomitant with the increased cell death in the leaves of *35S:UMAMIT14-4* plants (Figures 5D–G). These results suggest that over-expression of *UMAMIT14* disrupts amino acid homeostasis leading to a constitutive immune response against *Hpa*.

## Discussion

In the present study, it has been shown that over-expressing four independent UMAMITs that function as amino acid exporters causes a stunted growth phenotype (Figures 1, 2 and Supplementary Figure 1). Further studies of two such over-expressor lines, *35S:UMAMIT14-4* and *35S:UMAMIT14-6*, showed altered physiological traits such as a decrease in biomass and seed yield (Table 1). *UMAMIT14* over-expression also led to accumulation of SA and upregulation of a marker gene responding to the SA-dependent signaling pathway, *PR1* (Malamy et al., 1990; Van Loon and Van Strien, 1999). *ALD1* expression was also increased, indicating that the PIP/NHP-mediated pathway was also activated. Corroborating the activation of SA and PIP/NHP pathways, increased resistance to an SA-responsive pathogen was also observed (Figures 4, 5). Thus, a variety of elevated immune response hallmarks were observed.

The results from our study are in agreement with previous reports in which the mis-regulation of amino acid transport led to stress responses (Pilot et al., 2004; Hirner et al., 2006; Yang et al., 2014; Liu et al., 2016). Additionally, a forward genetics screen revealed that the *RESISTANCE TO PHYTOPHTHORA PARASITICA 1* (*RTP1*) mutant, which shows an elevated SA-mediated stress response, is caused by a defect in the *UMAMIT36* gene, although amino acid transport activity for UMAMIT36 has yet to be established (Pan et al., 2016). It is worthwhile noting that such defense-inducing phenotypes are likely associated with both increased (in case of *CAT1* over-expression and RTP1 loss-of-function) and decreased (*LHT1* knock-out, *GDU1-D, UMAMIT14, UMAMIT 18, UMAMIT 24, UMAMIT 25* over-expression) amino acid retention in the cytosol. In cases where amino acid profiles of the mutants were investigated, there was no clear common trend for the contents of specific amino acids being altered (Pilot et al., 2004; Hirner et al., 2006). Examination of amino acid profiles in 35S-UMAMIT14-4 line corroborates with these results, showing some amino acid composition changes but with no general trend common with *lht1* or *GDU1-D* mutants (Supplementary Figure 3). This is similar to the results from multiple studies reporting the amino acid contents in various mutants of amino acid biosynthesis genes (Van Damme et al., 2009; Hwang et al., 2011; Stuttmann et al., 2011; Alvarez et al., 2012). While all mutants showed enhanced defenses responses, they all displayed different amino acid accumulations and profiles, without any clear correlation between single amino acid contents or phenotypes.

The exact mechanism through which the SA-mediated defense pathway is triggered in these amino acid transporter or metabolic enzyme mutants is unknown. One potential pathway is via pipecolic acid (PIP) and its derivative, N-hydroxypipecolic acid (NHP). PIP and NHP are generated in the tissue attacked by biotrophic pathogens, and NHP functions as a long-distance signal to induce systemic resistance in distal leaves (Bernsdorff et al., 2016; Chen et al., 2018). PIP is generated from Lys; therefore a change in Lys metabolism might trigger an overproduction of PIP/NHP. In addition, PIP is also an analog of the proteogenic amino acid Pro. Currently there is no evidence showing that any of these amino acid transporters transport PIP/NHP at a physiologically relevant concentration. If they do function as PIP/NHP transporters, at least some of the phenotypes might be explained by mis-localization of these signaling molecules. Another potential pathway could be via GCN2; amino acid deficiency caused by transporter misregulation could increase the concentration of free tRNA, which is perceived by GCN2 to induce stress responses (Li et al., 2013). Additionally, the change in the concentration of extracellular amino acids might trigger the responses of GLRs (reviewed in Forde and Roberts, 2014). Deciphering the exact mechanism will be complex, because these pathways are interconnected. For example, PIP/NHP induces the production of SA (Navarova et al., 2012), which, in turn, induces GCN2-dependent elF2 phosphorylation (Lageix et al., 2008). Although the downstream components of GLRs are not well known, AtGLR3;3 seems to be required for defense responses against biotrophic pathogens, which is dependent on the SA pathway (Manzoor et al., 2013). Genetic experiments conferring altered transporter activities, defects in PIP/NHP synthesis, and/or defects in GCN2- or GLR-mediated signaling could help to elucidate the canonical components required for the immune responses triggered by misregulation of amino acid transporters.

SA is also known to interact with other plant hormones. For example, there is a well-characterized reciprocal antagonism between SA and JA pathways (Ikemura et al., 1990). SA also suppresses the function of auxin by downregulating the auxin receptor, TIR1 (Wang et al., 2007). MYB96, a transcription factor downstream of ABA signaling is also known to induce SA response, and activation tagging line *myb97-1* shows a dwarf phenotype which is dependent of SA production (Seo and Park, 2010). Therefore, it is possible that other hormones are involved in triggering the stress response observed in UMAMIT14 overexpressing plants. In our current study, however, we did not observe any consistent increase or decrease of other plant hormones (IAA, ABA, and JA) in the UMAMIT14 overexpression lines. Therefore, it is likely that the phenotypic responses we observe is primarily due to the activation of SA pathway.

While constitutive activation of the SA pathway via loss or gain of function of amino acid transport function to combat pathogens is unlikely to be an agronomically useful strategy due to the loss of biomass and seed yield, these characterized UMAMITs provide tools to study the link between amino acid levels and plant immune responses. For example, an inducible expression system of UMAMITs may be useful in identifying the early events triggered by enhanced amino acid export through RNAseq studies. Amino acids serve as the main N form acquired from the host in biotrophic pathogens, many of which induce accumulation of a specific set of amino acids at the infected site (Mckee et al., 1972; Kumar and Prasad, 1992; Horst et al., 2010a,b; Ward et al., 2010). Therefore, the pathway triggered by the overexpression of UMAMITs could represent the endogenous monitoring system for pathogen-induced alteration of amino acid metabolism. Positive regulation of SA pathway via amino acid export could interact with other SA-dependent defense mechanism activated in parallel, such as PAMP- and effector-triggered immunity (Pieterse et al., 2012). Indeed, a recent study revealed that amino acid transporter expression profiles in Arabidopsis leaves are different between compatible (i.e., the pathogen can establish an infection) and incompatible (i.e., the pathogen is incapable of infection) interactions, suggesting that amino acid transporter reprogramming is required to establish the infection (Sonawala et al., 2018). The significant question is whether the amino acid transporters are induced by the pathogens to support their growth, similar to the case of sugar exporters SWEET11 and 12 (Gebauer et al., 2017), or whether the plant actively regulates amino acid transporter activity to counter infection, as suggested by differential regulation of local amino acid transporters upon perception of pathogen-associated molecular patterns (PAMP) (Anderson et al., 2014) and systemic alteration of amino acid transporter activities in distal leaves (Schwachtje et al., 2018). Careful examination using genetics, physiology and molecular biology will be necessary to parse out such differences.

## Data Availability Statement

The raw data supporting the conclusions of this article will be made available by the authors, without undue reservation, to any qualified researcher.

## Author Contributions

JB, SO, JM, and GP designed the experiments. JB, US, BM, SF, and EC conducted experiments. JB, SO, SF, EC, and GP contributed in writing the manuscript. SO and GP supervised the project. All authors contributed to the article and approved the submitted version.

## Conflict of Interest

The authors declare that the research was conducted in the absence of any commercial or financial relationships that could be construed as a potential conflict of interest.
