# Supplementary material for: Increased Expression of UMAMIT Amino Acid Transporters Results in Activation of Salicylic Acid Dependent Stress Response
Source: Front Plant Sci. 2021 Jan 26;11:606386. doi: 10.3389/fpls.2020.606386 (PMC7870477; doi:10.3389/fpls.2020.606386)
Supplement: Supplementary file 1 [file Presentation_1.PPTX]

## Slide 1
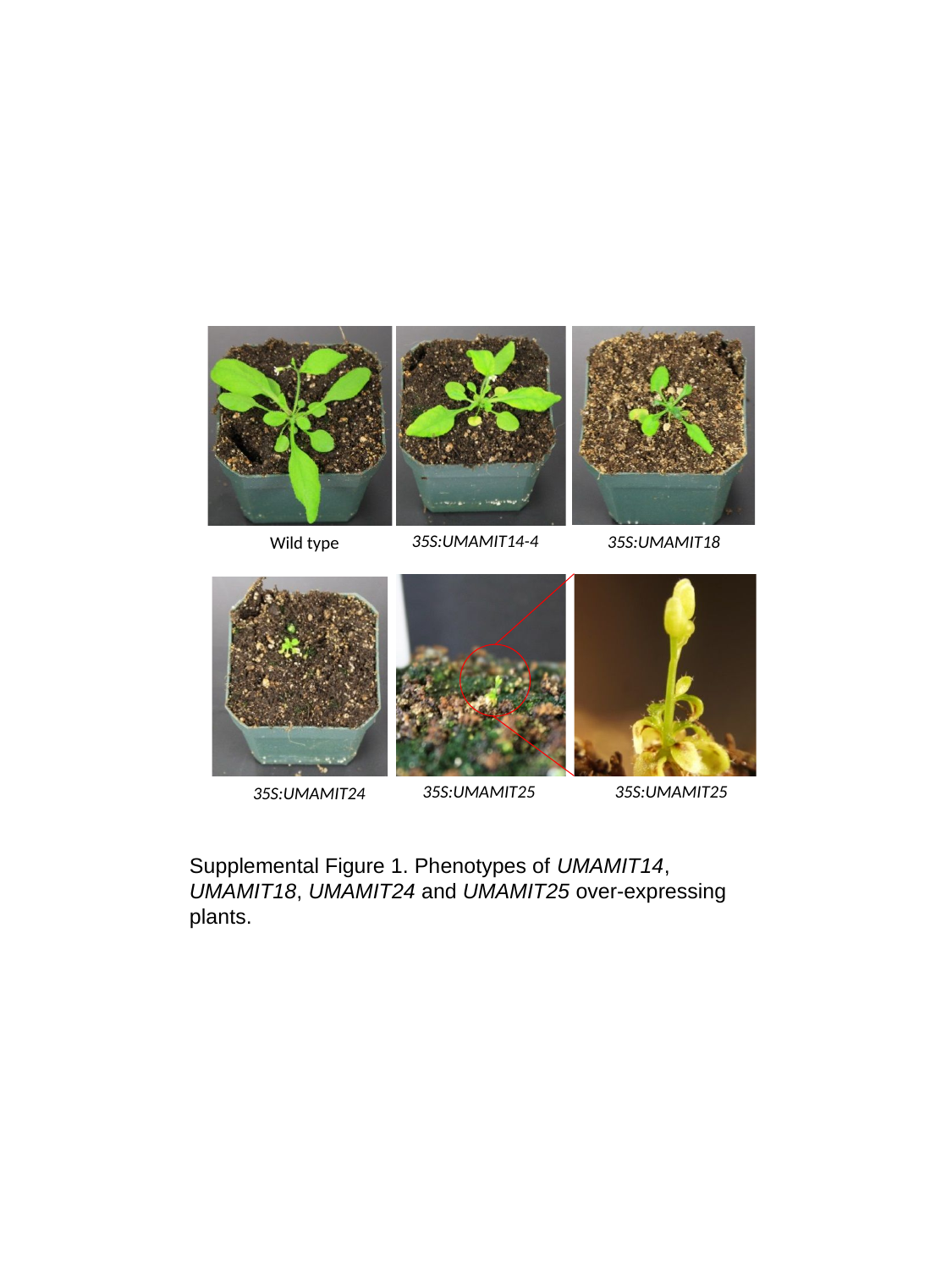

35S:UMAMIT14-4
35S:UMAMIT18
Wild type
35S:UMAMIT25
35S:UMAMIT25
35S:UMAMIT24
Supplemental Figure 1. Phenotypes of UMAMIT14, UMAMIT18, UMAMIT24 and UMAMIT25 over-expressing plants.

## Slide 2
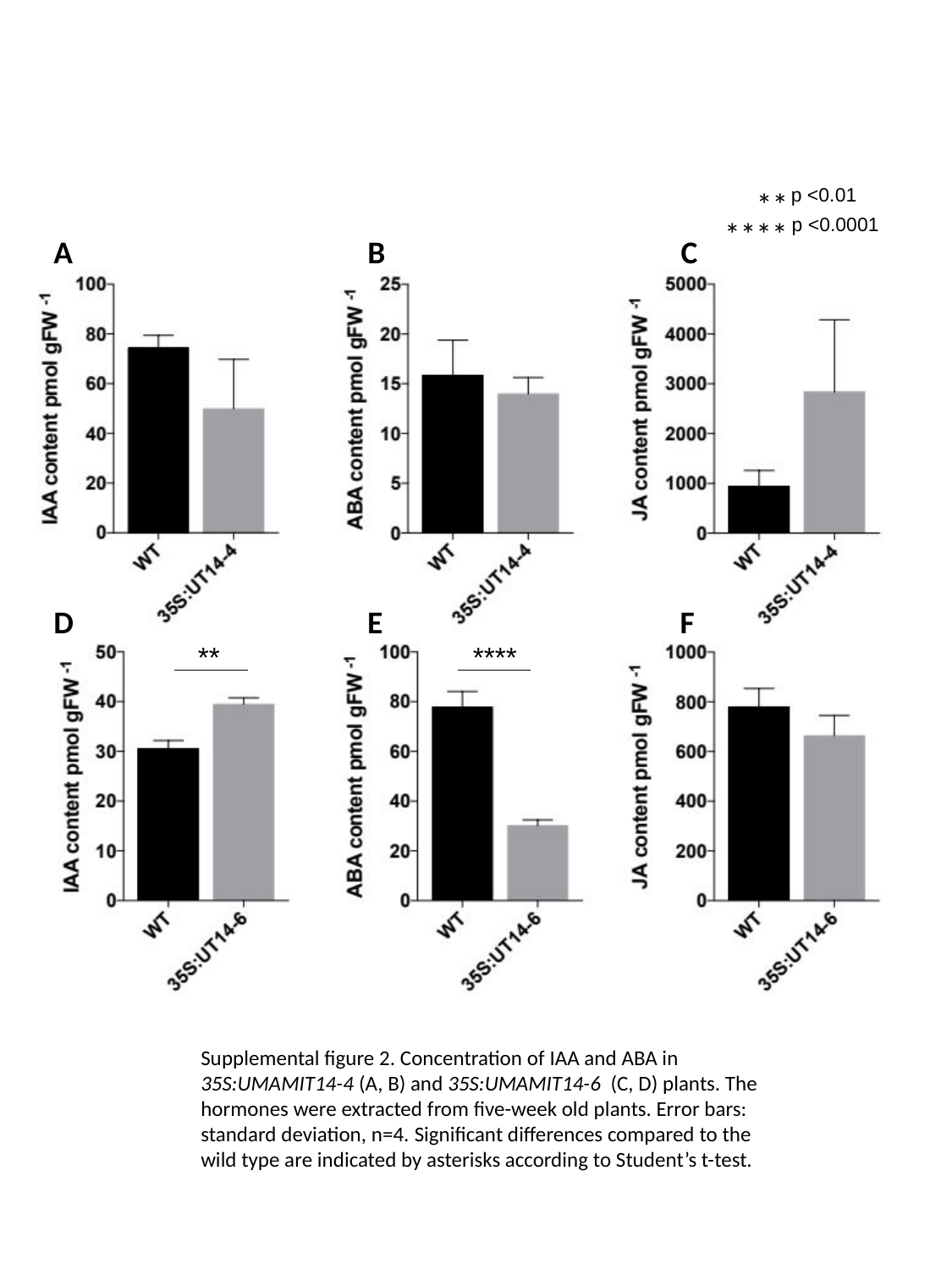

p <0.01
**
p <0.0001
****
A
B
C
D
E
F
**
****
Supplemental figure 2. Concentration of IAA and ABA in 35S:UMAMIT14-4 (A, B) and 35S:UMAMIT14-6 (C, D) plants. The hormones were extracted from five-week old plants. Error bars: standard deviation, n=4. Significant differences compared to the wild type are indicated by asterisks according to Student’s t-test.

## Slide 3
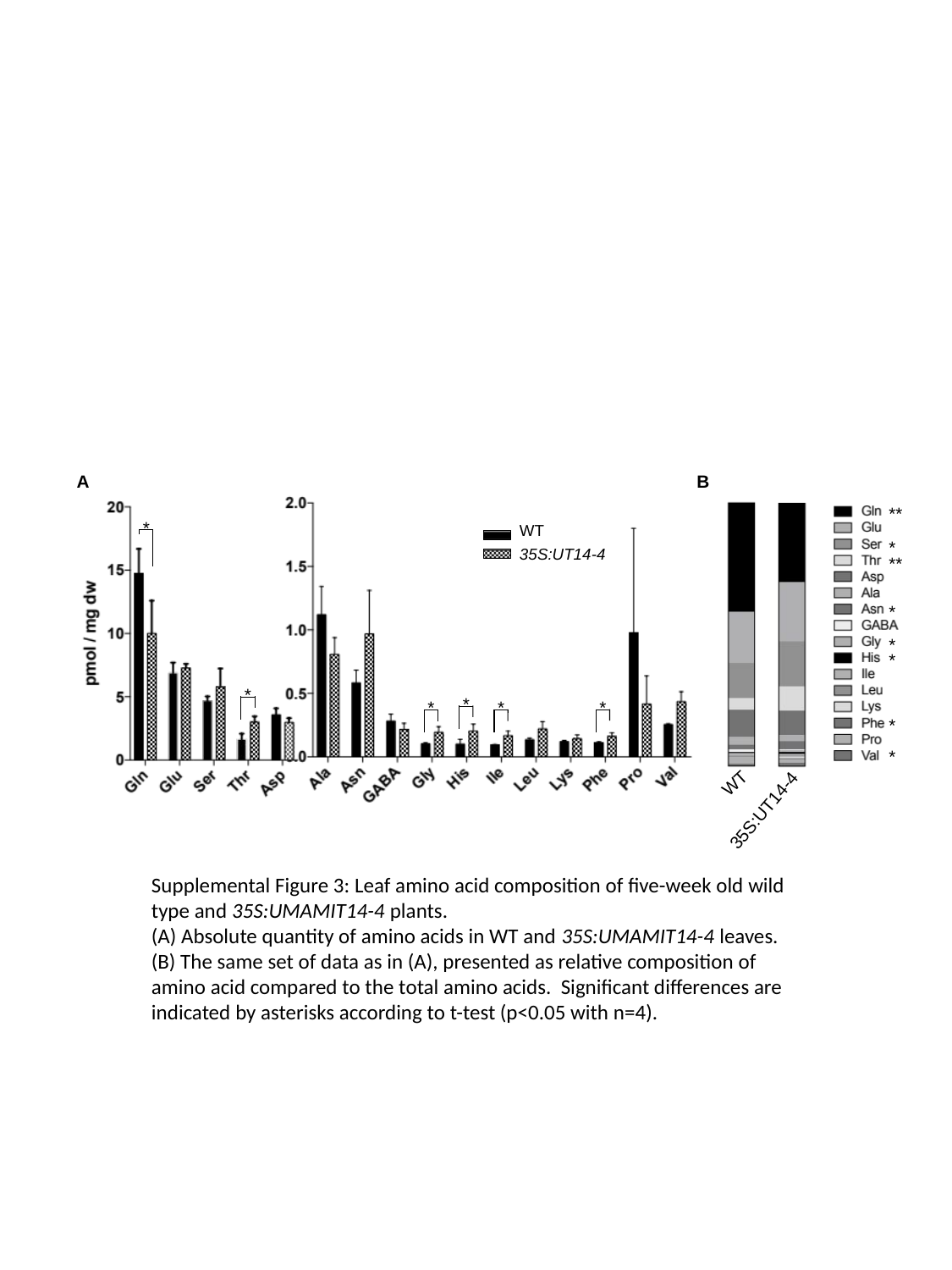

#
A
B
**
*
WT
*
35S:UT14-4
**
*
*
*
*
*
*
*
*
*
*
WT
35S:UT14-4
Supplemental Figure 3: Leaf amino acid composition of five-week old wild type and 35S:UMAMIT14-4 plants.
(A) Absolute quantity of amino acids in WT and 35S:UMAMIT14-4 leaves. (B) The same set of data as in (A), presented as relative composition of amino acid compared to the total amino acids. Significant differences are indicated by asterisks according to t-test (p<0.05 with n=4).

## Slide 4
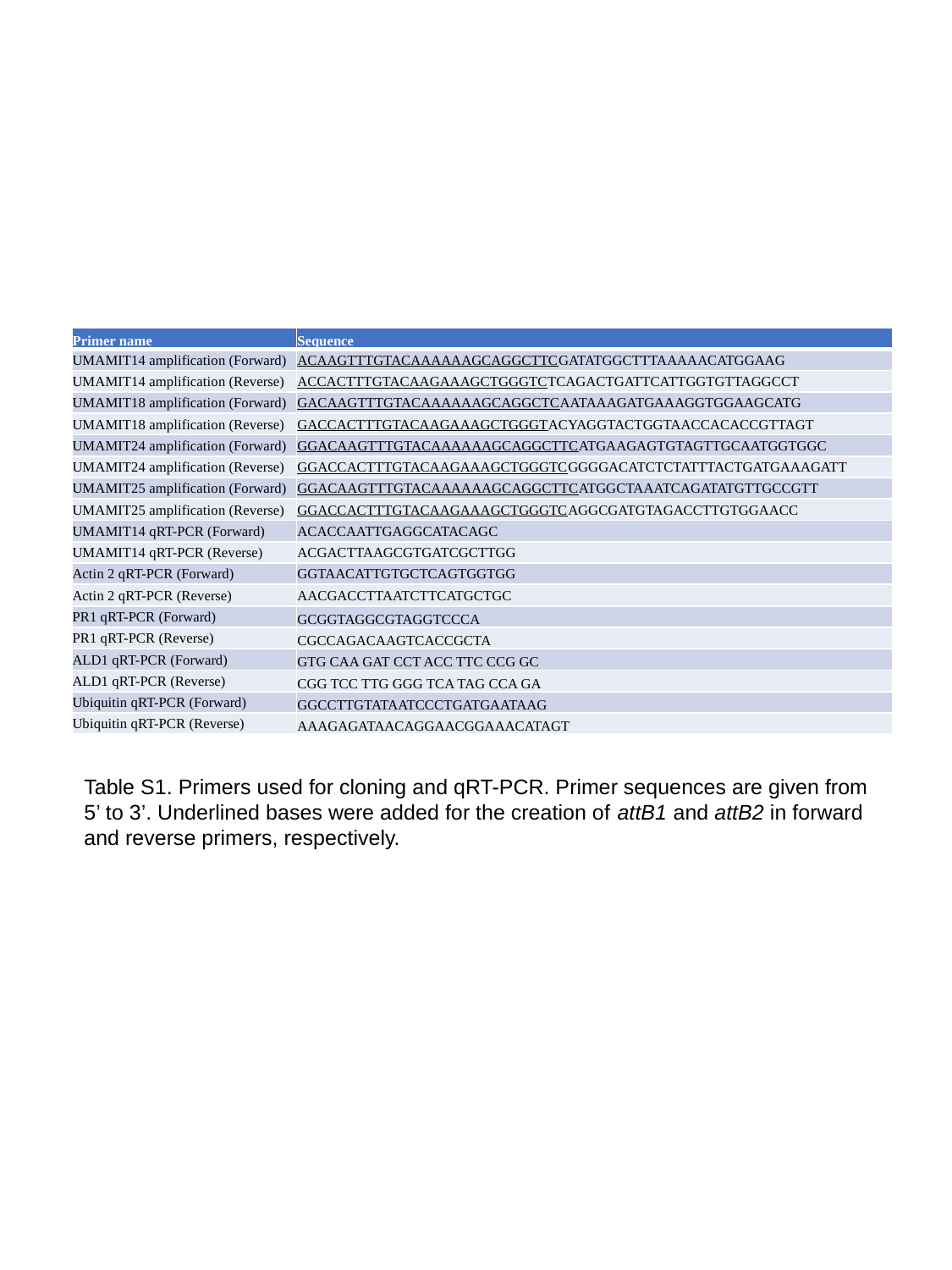

| Primer name | Sequence |
| --- | --- |
| UMAMIT14 amplification (Forward) | ACAAGTTTGTACAAAAAAGCAGGCTTCGATATGGCTTTAAAAACATGGAAG |
| UMAMIT14 amplification (Reverse) | ACCACTTTGTACAAGAAAGCTGGGTCTCAGACTGATTCATTGGTGTTAGGCCT |
| UMAMIT18 amplification (Forward) | GACAAGTTTGTACAAAAAAGCAGGCTCAATAAAGATGAAAGGTGGAAGCATG |
| UMAMIT18 amplification (Reverse) | GACCACTTTGTACAAGAAAGCTGGGTACYAGGTACTGGTAACCACACCGTTAGT |
| UMAMIT24 amplification (Forward) | GGACAAGTTTGTACAAAAAAGCAGGCTTCATGAAGAGTGTAGTTGCAATGGTGGC |
| UMAMIT24 amplification (Reverse) | GGACCACTTTGTACAAGAAAGCTGGGTCGGGGACATCTCTATTTACTGATGAAAGATT |
| UMAMIT25 amplification (Forward) | GGACAAGTTTGTACAAAAAAGCAGGCTTCATGGCTAAATCAGATATGTTGCCGTT |
| UMAMIT25 amplification (Reverse) | GGACCACTTTGTACAAGAAAGCTGGGTCAGGCGATGTAGACCTTGTGGAACC |
| UMAMIT14 qRT-PCR (Forward) | ACACCAATTGAGGCATACAGC |
| UMAMIT14 qRT-PCR (Reverse) | ACGACTTAAGCGTGATCGCTTGG |
| Actin 2 qRT-PCR (Forward) | GGTAACATTGTGCTCAGTGGTGG |
| Actin 2 qRT-PCR (Reverse) | AACGACCTTAATCTTCATGCTGC |
| PR1 qRT-PCR (Forward) | GCGGTAGGCGTAGGTCCCA |
| PR1 qRT-PCR (Reverse) | CGCCAGACAAGTCACCGCTA |
| ALD1 qRT-PCR (Forward) | GTG CAA GAT CCT ACC TTC CCG GC |
| ALD1 qRT-PCR (Reverse) | CGG TCC TTG GGG TCA TAG CCA GA |
| Ubiquitin qRT-PCR (Forward) | GGCCTTGTATAATCCCTGATGAATAAG |
| Ubiquitin qRT-PCR (Reverse) | AAAGAGATAACAGGAACGGAAACATAGT |
Table S1. Primers used for cloning and qRT-PCR. Primer sequences are given from 5’ to 3’. Underlined bases were added for the creation of attB1 and attB2 in forward and reverse primers, respectively.
